# Supplementary material for: Muscle-Specific Effects of Genotype, Animal Age, and Wet Aging Duration on Beef Color, Tenderness, and Sensory Characteristics
Source: Animals (Basel). 2024 Dec 12;14(24):3593. doi: 10.3390/ani14243593 (PMC11672512; doi:10.3390/ani14243593)
Supplement: Supplementary file 1 [file animals-14-03593-s001.zip › animals-3342649-supplementary.pdf]

Supplementary Table 1. Effects of bull type, and age on live weight, hot carcass weight, backfat thickness, and ribeye area of humped and humpless bulls

Carcass characteristics	Bull type		Bull age (months)		SE	P-value	
	Humped	Humpless	21±2	30±3		Bull type	Bull age
LW (kg)	278.8	303.7	246.4	336.2	4.55	< 0.001	< 0.001
HCW (kg)	139.9	151.3	122.6	168.6	2.70	0.003	< 0.001
Backfat thickness (mm)	2.4	2.5	2.4	2.5	0.15	NS	NS
Ribeye area (sq cm)	35.3	33.2	32.3	38.5	0.25	NS	0.005

Note: LW= Live weight, HCW= Hot carcass weight, SE= Standard error, NS= P>0.05, * = P<0.05, ** = P<0.01, *** = P<0.001

Supplementary Table 2: Pearson correlation analysis among various meat quality characteristics.

	pH	L*	a*	b*	C*	DL%	CL%	WBSF	TBARS	Sensory Juiciness	Sensory Flavor	Sensory Tenderness	Sensory Overall acceptability
**L***	-0.593												
	0.001												
**a***	-0.721	0.751											
	0.000	0.000											
**b***	0.136	-0.329	-0.275										
	0.227	0.003	0.013										
**C***	-0.668	0.706	0.795	-0.171									
	0.001	0.000	0.000	0.129									
**DL%**	-0.691	0.718	0.744	-0.314	0.796								
	0.001	0.000	0.000	0.005	0.000								
**CL%**	-0.712	0.244	0.484	-0.119	0.449	0.469							
	0.000	0.029	0.001	0.294	0.002	0.002							
**WBSF**	0.070	0.013	0.128	-0.315	0.012	-0.085	-0.045						
	0.536	0.907	0.256	0.004	0.915	0.453	0.689						
**TBARS**	0.638	-0.595	-0.605	0.595	-0.499	-0.612	-0.459	-0.206					
	0.000	0.000	0.000	0.000	0.002	0.000	0.001	0.067					
**Sensory Juiciness**	0.059	-0.062	-0.05	0.210	-0.055	-0.043	0.099	-0.137	0.201				
	0.604	0.582	0.66	0.061	0.626	0.704	0.382	0.226	0.074				
**Sensory Flavor**	-0.008	0.123	0.097	-0.239	-0.019	-0.012	-0.076	0.172	-0.132	-0.234			
	0.944	0.276	0.393	0.033	0.869	0.913	0.502	0.126	0.245	0.037			
**Sensory Tenderness**	-0.293	0.037	-0.005	0.290	0.111	0.228	0.285	-0.803	0.044	0.184	-0.238		
	0.008	0.748	0.965	0.049	0.325	0.042	0.010	0.000	0.701	0.103	0.034		
**Sensory Overall acceptability**	-0.243	0.154	0.08	0.100	0.022	0.096	0.240	-0.212	-0.053	0.221	-0.014	0.319	
	0.030	0.171	0.479	0.377	0.844	0.399	0.032	0.059	0.640	0.049	0.903	0.004	

Contents:
Pearson correlation, P-Value
L*= Lightness, a*= redness, b*= yellowness, C*= Chroma, DL%= Drip loss percentage, CL%= Cooking loss percentage, WBSF= Warner Bratzler shear force , TBARS= Thiobarbituric Acid Relative Substances
